# Does the media (also) keep the score? Media-based exposure to the Russian-Ukrainian war and mental health in Portugal

**DOI:** 10.1177/13591053231201242

**Published:** 2023-10-15

**Authors:** Marta Castro, Joana Aires Dias, Luis Madeira

**Affiliations:** 1 Faculdade de Medicina, Universidade de Lisboa, Portugal; 2 ICNAS/CIBIT, Faculdade de Medicina, Universidade de Coimbra, Portugal; 3 CUF Descobertas Hospital, Portugal

**Keywords:** acute, adaptation, post-traumatic, psychological, psychological trauma, stress, stress disorders, traumatic

## Abstract

The Russian-Ukrainian war (RUW) is responsible for extensive individual suffering and a socio-economic impact on the world and is reshaping global affairs. Many studies have focused on direct exposure to conflict and several public health policies have been devised. Nonetheless, indirect exposure through media has received minimal attention and there is limited evidence that mental health symptoms and disorders may arise as a result. We explored the role of voluntary or involuntary media-based exposure to the RUW on individuals’ mental health including stress symptoms, coping strategies, daily functioning, and worries across demographic variables. In our sample, subjects with involuntary and higher amount of exposure seem to have higher stress symptoms. Also, those who had previous ruminations on war issues could be at risk of developing more post-traumatic stress symptoms. Therefore, media appears to be a conduit that spreads negative consequences of community trauma beyond directly affected communities.

## Introduction

On 24th February 2022, one of the largest and fastest-growing humanitarian emergencies since World War II: the Russian-Ukrainian conflict, began. Since then, according to the United Nations Refugee Agency (UNHCR, 2023), over 8.2 million Ukrainian people have been displaced, and an extensive socio-economic impact has been felt worldwide. Trauma is an inevitable consequence of war and exposure to trauma during armed conflict has an enormous potential to harm mental health, which can lead to high rates of Post-Traumatic Stress Disorders (PTSD), and those who are directly exposed to the Russian-Ukraine conflict are especially susceptible (Johnson et al., 2022). Contrary to early world conflicts, wars nowadays are widely publicized. Individuals have access to direct images and videos of the gruesome events taking place in Ukraine. Exposure to images of trauma has been linked to the trauma itself as this may in itself be a cause of stress, psychopathological symptoms and/or mental disorders, which are important public health issues. Features of the coverage (media form, coverage content) and the extent of an individual’s contact with said coverage may be a determinant of stress-related outcomes (Propper et al., 2007). Indeed, according to Brewin (1993), ‘television is the worst offender because the visual impact is unforgettable, and any reasonable sense of proportion goes out of the window’.

People live in a time where man-made conflicts are no longer confined within a geographical place, and they almost immediately accept the suffering taking place in other parts of the world from the comfort of their homes and workplaces. On September 11, 2001, television transmitted dramatic images of planes crashing into the World Trade Center and people jumping from buildings, in real-time. The event and subsequent conflict received considerable media coverage and the impact was so dramatic that it determined new policies for media coverage (Vasterman et al., 2005). The SARS-CoV-2 global crisis media coverage exacerbated mental health issues, particularly through the rapid spread of dramatic events and the widespread disinformation that ensued (Su et al., 2021). Furthering this claim, this pandemic might not only have exacerbated the reliance on the internet, further cementing the hold on media sources, but it has also caused several stresses, which may have worsened overall mental health, thus making humans more vulnerable (Su et al., 2023). It has been estimated that there are an additional 76.2 million cases of anxiety disorders and 49.4 million major depressive disorders globally, due to the COVID-19 pandemic (Santomauro et al., 2021).

Images of trauma passed on through the media seem to be the cause of several pathological symptoms. The most obvious of those is their role in desensitization and numbing, leading to depersonalization/derealization phenomena and/or callous traits where viewers get used to traumatic images and their empathy is reduced. Nonetheless, media-based exposures to trauma have been shown to lead to anxious (Cardeña et al., 2008; Dixon et al., 1993) and depressive symptoms (Riad et al., 2022), and generally determine adverse physical and mental health outcomes such as Post-Traumatic Stress Disorder (PTSD) (Holman et al., 2008). A previous study, post 9/11 attacks, with a population composed mostly of people exposed to this event only by watching television, enhanced the importance of considering adverse physical and mental health outcomes in this group, by suggesting that chronic reminders may prolong the physiological arousal, rendering them more vulnerable to cardiovascular ailments (Holman et al., 2008).

There is a large body of evidence supporting the role of media in stress, including a positive correlation between the extent of exposure and stress levels and many forms of adverse events. Data also show that witnessing trauma-related stories via the media may prolong previous trauma and encourage ruminative thinking (Holman et al., 2014), which may lead the subject to dwell on prior negative events (Holman and Silver, 1998). Indeed, in such a state of activation of fear, circuits in the brain play an important role in developing PTSD responses to stress. Surprisingly, evidence suggests that indirect exposure to collective trauma through the media can lead to higher acute stress symptoms than all forms of direct exposure (Goodwin et al., 2013; Holman et al., 2014; Silver et al., 2013). Despite growing evidence of its role in prolonging trauma (Holman et al., 2014), media-based exposure has not yet been considered an official trigger or exposure criterion for PTSD (American Psychiatric Association, 2013).

Trauma seems, therefore, to be spreading beyond directly affected communities to individuals who are repeatedly engaging with its media content, sometimes for several hours a day. The present study aims to explore the effect that previous media-based exposure to recent war events is having on the general population in Portugal, by analysing (1) the role of involuntariness and the amount of exposure to war-media in the expression of stress symptoms; (2) how acute stress symptoms and overall functioning could differ in those with more concerns about the war and fear about the future; and (3) its impact on physical and psychological health-related outcomes across demographic and clinical features.

In this study, the first hypothesis is that voluntary (e.g. search for war news and updates) or involuntary (e.g. access to information in social media without search for it) contact with media coverage of the Russian-Ukrainian conflict may determine stress symptoms in individuals not directly affected by the war. The second hypothesis is that those with more concerns about the Russian-Ukrainian war reveal more stress symptoms, which disrupts their daily functioning and leads to more worries about the future. Finally, the third hypothesis is that a higher amount of exposure could lead to more distress and fear. Also, this study explores some coping strategies, overall functioning and the role of age and gender on the levels of fear of the future and stress.

Understanding how widespread media exposure to trauma events may play a role in individuals’ well-being might allow governing bodies to better regulate media.

## Methods

### Data collection

This cross-sectional study was conducted using an online questionnaire (Google Forms^®^) between November 2022 and January 2023. Advertising occurred through the Portuguese Psychiatry Association and Medical Student Association of Lisbon University public online social networks (Facebook^®^, Instagram^®^ and LinkedIn^®^). All data were anonymized, written informed consent (Helsinki declaration adjusted) was gathered from all the participants and Ethical approval was provided by CAML Ethics Committee (Lisbon Academic Medical Centre; Ethics Committee Federal Wide Assurance Number 87/22) in November 2022.

### Participants and procedure

This research included 213 adult participants fluent in Portuguese, capable of giving informed consent. Data on sociodemographic and clinical variables included age, gender, employment, place of residency, marital status, history of psychiatry disorder, alcohol and drug consumption. Participants responded to four self-report measures, including *The Primary Care PTSD Screen for DSM-5* (PC-PTSD-5) (Prins et al., 2016), *The MOS 36-item short-form health survey* (SF-36) (Ware and Sherbourne, 1992), Portuguese version (Ferreira, 2000a), *Brief-COPE* (Carver, 1997), Portuguese version (Pais Ribeiro and Rodrigues, 2004) and *Stanford Acute Stress Reaction Questionnaire* (SASRQ) (Cardeña et al., 2000), Portuguese version (Pires, 2021). Participants also responded to two items assessing ongoing worries about terrorism (Holman et al., 2008) (Fear of the Future). Portuguese-validated versions of the instruments were used except for the PC-PTSD-5, and Fear of the Future item, which were subjected to a cross-cultural team translation approach, supported by recent evidence (Behr and Braun, 2023).

### Measures and instruments

(1) *The Primary Care PTSD Screen for DSM-5* (Prins et al., 2016)

This is a five-item screening tool, in a dichotomous (yes/no) format, that has been validated against clinician diagnostic interviews. This measure also includes an initial yes/no question about the possibility of the individuals having experienced a traumatic event. The main objective is to screen PTSD, according to the *DSM-5*, questioning post-traumatic symptoms in the previous month. The minimum rating is 0 and the maximum is 5. As recommended by Prins et al. (2016), an optimal cut-off of 4 was used to determine possible PTSD diagnosis.

(2) *The MOS 36-item short-form health survey* (Ware and Sherbourne, 1992)

This is a 36-item tool modified from the SF-36 (Portuguese-validated version by Ferreira, 2000b). This scale evaluates eight health concepts: (1) physical functioning (min. = 10, max. = 30; α = 0.86), (2) role limitations due to physical health problems (min. = 4, max. = 20; α = 0.74), (3) bodily pain (min. = 2, max. = 11; α = 0.85), (4) social functioning (min. = 2, max. = 10; α = 0.29), (5) general mental health (min. = 5, max. = 25; α = 0.83), (6) role limitations due to emotional health problems (min. = 4, max. = 20; α = 0.69), (7) vitality (min. = 4, max. = 20; α = 0.78), (8) general health perceptions (min. = 5, max. = 25; α = 0.79). (1) is a 3-point scale (1 = very limited, 3 = no limitations) and all the others are 5-point scales (1 = never, 5 = always).

(3) *BRIEF COPE* (Carver, 1997)

This is a 28-item multidimensional measure of strategies used for coping in response to stressors. This abbreviated inventory is comprised of items that assess the frequency with which a person uses different coping strategies rated on a scale from 0 = I haven’t been doing this at all, to 3 = I’ve been doing this a lot. In this study, the Portuguese-validated version was used (Pais Ribeiro and Rodrigues, 2004). There are 14 two-item subscales within the Brief COPE, analysed separately: (1) self-distraction (α = 0.67), (2) active coping (α = 0.65), (3) denial (α = 0.72), (4) substance use (α = 0.81), (5) use of emotional support (α = 0.79), (6) use of instrumental support (α = 0.81), (7) behavioural disengagement (α = 0.78), (8) venting (α = 0.84) (9) positive reframing (α = 0.74), (10) planning (α = 0.70), (11) humour (α = 0.83), (12) acceptance (α = 0.55), (13) religion (α = 0.80) and (14) self-blame (α = 0.62). The minimum rating for each is 0 and the maximum is 6.

(4) *Stanford Acute Stress Reaction Questionnaire* (Cardeña et al., 2000)

This is a measure of acute stress symptoms, used to report how often subjects experienced 30 possible acute posttraumatic reactions in the previous month and the aftermath on a 5-point scale from 0 (not experienced) to 5 (experienced very often). The minimum rating is 0 and the maximum is 150. In this study, the Portuguese-version was used (Pires, 2021) (α = 0.93).

(5) Two items to assess ongoing worries about terrorism from a previous study (Holman et al., 2008): ‘In the past week, how often have you had fears about the possibility of another terrorist attack (bioterrorism, hijacking, etc.)?’, ‘I worry that an act of terrorism (bioterrorism, hijacking, etc.) will personally affect me or someone in my family in the future’. Items were scored on a 5-point Likert scale (1 = never, 5 = all the time), analysed separately. The minimum rating is 1 and the maximum is 5.

### Statistical analysis

Statistical analysis was performed using *Statistical Package for the Social Sciences* (SPSS^®^) v.27. Descriptive statistics were performed to characterize demographic and clinical variables, and measures (including internal consistency according to Cronbach’s alpha). Before hypothesis testing, a Kolmogorov-Smirnov normality test and histograms were produced to assess normality. The null hypothesis was rejected for all scales (*p* < 0.05) and thus this study relied on non-parametric tests (i.e. Mann-Whitney *U*, Kruskal-Wallis H with Bonferroni correction for multiple comparisons). When possible, exact *p*-value is shown in alternative to the asymptotic *p*-value. To understand the effect that concerns about the war in Ukraine had on measures, a comparison was made between the (1) group who referred to the war as the most stressful event of the previous month and the (2) group who reported not having had any stressful events (control group). For other comparisons, the sample was divided into three groups: the (1) group who reported being exposed to war-media voluntarily, the (2) group who reported involuntary exposure and the (3) group who reported being exposed both voluntarily and involuntarily. The SF-36 ‘Body-pain’ subscale was not considered in analysis, following an item excluded prior to the release of the questionnaire, that could not be included afterwards.

## Results

### Sociodemographic and clinical data

This sample was on average 36.8 years-old (Sd = 16.5, Mdn = 30.0, range: 18–85) and equally distributed across genders (54.5% female). More than half were employed (54.7%) and lived in the Lisbon and Tagus Valley region (58.5%). Considering the use of drugs, it was noted that 52.6% have never smoked, while the majority used alcohol socially (83.1%) and only 19.3% use or have used other drugs. Twenty-eight participants (13.3%) reported a present or past psychiatric diagnosis, depression being the most common (50.0%). Three participants did not report on several sociodemographic variables, and so were not included in analyses involving these variables. Detailed sample’s data is presented in the Supplemental Material.

### Psychological measures

A first analysis was conducted to obtain descriptive statistics on all psychological measures. Alpha values ranged from very poor (α = 0.31) to excellent (α = 0.97), with the Brief-COPE subscales obtaining lower values. Alpha was not calculated for the SF-36’s Changes in Health subscale nor the two individual questions pertaining to Fear of the Future, due to them being individual items. All measures’ descriptive statistics can be found in Table 1.

**Table 1.** Psychological measures’ descriptive statistics and alpha values.

Scale	*M* (Sd)	Mdn (range)	α
Brief-COPE			
Active coping	4.7 (1.3)	5.0 (0–6)	0.68
Planning	3.7 (1.5)	4.0 (0–6)	0.66
Instrumental support	1.8 (1.7)	1.0 (0–6)	0.65
Emotional support	2.7 (1.6)	2.0 (0–6)	0.53
Religion	1.9 (1.9)	1.0 (0–6)	0.88
Positive reframing	3.8 (1.5)	4.0 (0–6)	0.60
Self-blame	2.9 (1.5)	3.0 (0–6)	0.66
Acceptance	4.0 (1.4)	4.0 (0–6)	0.60
Venting	2.7 (1.4)	3.0 (0–6)	0.31
Denial	1.4 (1.4)	1.0 (0–6)	0.48
Self-distraction	4.1 (1.4)	4.0 (0–6)	0.47
Behavioural disengagement	1.1 (1.3)	1.0 (0–5)	0.60
Substance use	0.3 (0.8)	0.0 (0–6)	0.88
Humour	3.1 (1.9)	3.0 (0–6)	0.84
PC-PTSD-5	0.5 (1.1)	0.0 (0–5)	0.70
SASRQ	42.8 (34.5)	35.0 (0–150)	0.97
Re-experience	8.0 (7.4)	6.0 (0–30)	0.89
Avoidance	9.7 (8.1)	8.0 (0–30)	0.90
Arousal	10.5 (7.8)	9.0 (0–30)	0.88
Deterioration of function	2.8 (2.8)	2.0 (0–10)	0.72
Dissociation	11.8 (11.3)	9.0 (0–50)	0.92
SF-36			
Physical functioning	91.3 (15.6)	95.0 (0–100)	0.90
Role limitations due to physical problems	82.5 (21.9)	93.8 (0–100)	0.93
General health perceptions	69.3 (18.0)	71.0 (0–100)	0.75
Vitality	53.8 (19.1)	56.3 (0–93.8)	0.80
Social functioning	74.8 (21.5)	75.0 (0–100)	0.81
Role limitations due to emotional problems	72.1 (26.8)	75.0 (0–100)	0.95
General mental health	66.3 (18.5)	70.0 (20–100)	0.83
Changes in health	40.7 (23.1)	50.0 (0–100)	–
Fear of the future			
Proximity of war	1.5 (0.7)	1.0 (1–5)	–
Effects of war on self or family	2.3 (1.0)	2.0 (1–5)	–

### PC-PTSD-5

Following the cut-off recommended (Prins et al., 2016), nine participants (4.2%) scored 4 or higher, which is relevant for diagnosing PTSD.

### SASRQ

When reporting on the most stressful event of the previous month, 22.5% (*n* = 48) participants reported not having experienced any stressful event (control group), while 20 (9.4%) participants referred to the war in Ukraine as the most stressful event experienced in the previous month. The later had a mean disturbance of 3.6 (Sd = 1.1, Mdn = 3.6, range: 1–5), and while 22.2% (*n* = 4) experienced stress-related symptoms for more than 5 days, the same percentage reported experiencing them for only 1 day.

### SF-36

While it was not possible to obtain a value for the Bodily Pain SF-36 subscale, it is possible to calculate descriptive statistics for the item ‘How much *bodily* pain have you had during the past month?’. The mean sample reported having low levels of pain (*M* = 1.8, Sd = 0.8, Mdn = 2.0, range: 1–4).

### Media exposure

Lastly, participants were asked to indicate whether exposure was voluntary, involuntary or mixed, and to estimate how many hours per day in the previous month they had been exposed to war-media. Most of the sample (48.8%, *n* = 104) referred to mixed exposure, while 33.3% (*n* = 71) and 17.8% (*n* = 38) referred to involuntary and voluntary exposure, respectively. As the amount of exposure/day was an open-ended question, 39 participants (18.3%) did not provide an estimate, and nine (4.2%) presented extreme results, being classified as extreme outliers (Tukey, 1977). These were excluded from the analyses. The remaining participants (*n* = 165) reported a mean of 1.1 hours of exposure (Sd = 1.1, Mdn = 1.0, range: 0–6).

## Group comparisons

### Sociodemographic and clinical factors

There were gender-wise differences with higher scores in female participants in (1) re-experiencing stress responses (*U* = 4700.00, *p* = 0.038) (SARSQ, Re-Experience), (2) level of disturbance due to the most stressful event (*U* = 4409.00, *p* = 0.005) (SARSQ), (3) use of self-distraction as a coping strategy (*U* = 4321.50, *p* = 0.003) (BRIEF-COPE, Self-Distraction) and (4) fear of the consequences for themselves or their family (*U* = 4686.00, *p* = 0.016) (Fear of the Future). In contrast, male participants had higher scores on physical (*U* = 4467.50, *p* = 0.006) and social (*U* = 4653.50, *p* = 0.027) functioning, vitality (*U* = 4207.00, *p* = 0.001), and general mental health (*U* = 4646.00, *p* = 0.028) (SF-36 subscales).

Age was divided into three groups: (1) 18–24, (2) 25–50 and (3) 51–85. Differences were also found when comparing age groups (Bonferroni correction applied). Regarding acute stress responses, the older group (group 3) had lower scores than the other two groups in the SASRQ full scale (*H*(2) = 16.76, *p* < 0.001) and subscales (*p* < 0.05). A similar pattern was found in the coping strategy of securing instrumental (*H*(2) = 20.33, *p* < 0.001) and emotional (*H*(2) = 54.51, *p* < 0.001) support, use of humour (*H*(2) = 16.1, *p* < 0.001) and making less use of self-blame (*H*(2) = 14.72, *p* < 0.001) (BRIEF-COPE subscales). The younger (group 1) and older group also differed in self-distraction-reported behaviours (*H*(2) = 8.01, *p* = 0.018), behavioural disengagement (*H*(2) = 9.39, *p* = 0.009) and substance use (*H*(2) = 12.09, *p* = 0.002) (BRIEF-COPE subscales), with younger participants holding higher values. When analysing health outcomes, results indicate that the younger group has increased physical functioning (*H*(2) = 12.92, *p* = 0.002), and vitality (*H*(2) = 29.68, *p* < 0.001) (SF-26 subscales) when compared to the other two groups. On the other hand, the older group scored higher on social functioning (*H*(2) = 21.20, *p* < 0.001), general mental health (*H*(2) = 11.34, *p* = 0.003) and on role limitations due to emotional problems (*H*(2) = 17.12, *p* < 0.001), the last indicating fewer role limitations (SF-26 subscales). Additionally, the older group spent more time than the younger group watching news of the war in Ukraine (*H*(2) = 11.97, *p* = 0.003).

### Hypothesis testing

Differences were found between those that considered the war the most stressful event and the control group, scoring higher in PC-PTSD-5 (*U* = 331.50, *p* = 0.007), and the use of instrumental support (*U* = 327.00, *p* = 0.033), and self-blame (*U* = 248.00, *p* = 0.001) as coping strategies (BRIEF-COPE subscales). Complementarily, the control group had higher scores of social functioning (*U* = 326.00, *p* = 0.031), general mental health (*U* = 281.50, *p* = 0.007), fewer limitations due to physical (*U* = 334.00, *p* = 0.036) and emotional (*U* = 293.00, *p* = 0.009) problems (SF-36 subscales), and was less fearful that war could happen near them (*U* = 176.00, *p* < 0.001) or that it might impact them or someone close to them (*U* = 233.50, *p* < 0.001) (Fear of the Future). Results comparing the two groups are found in Table 2.

**Table 2.** Statistical differences: group disturbed by the RUW and control group; three types of exposure to war-news.

Scales	Mean Ranks		Mann-Whitney U		Mean Ranks			Kruskal-Wallis H	
	None	War on Ukraine	*U*	*Exact-p*	I	V	V&I	*H*	*p*
Brief-COPE									
Active coping	34.4	34.9	473.00	0.927	100.4	107.1	111.5	1.50	0.473
Planning	31.8	41.0	351.00	0.077	113.7	102.8	104.0	1.31	0.518
Instrumental support	31.3	42.2	327.00	0.033	106.5	98.6	112.6	3.10	0.212
Emotional support	33.2	37.7	417.00	0.393	113.0	86.1	110.5	5.62	0.060
Religion	33.5	36.8	343.00	0.517	108.3	91.2	111.9	3.38	0.185
Positive reframing	34.3	35.1	469.00	0.884	113.6	90.3	108.6	3.83	0.147
Self-blame	29.7	46.1	248.00	0.001	114.8	103.9	102.9	1.76	0.414
Acceptance	34.7	34.0	470.50	0.899	111.3	109.7	103.1	0.87	00.647
Venting	32.8	38.6	398.00	0.262	117.2	107.1	100.0	3.41	0.181
Denial	31.7	41.2	345.50	0.060	120.9	111.8	95.8	7.82	0.020
Self-distraction	32.7	38.9	392.50	0.233	109.6	95.1	109.6	1.80	0.408
Behavioural disengagement	31.9	40.8	355.00	0.068	117.7	101.5	101.7	3.61	0.164
Substance use	34.4	34.8	474.00	1.000	112.2	113.4	101.2	4.77	0.092
Humour	34.3	34.9	472.50	0.920	108.7	119.0	101.4	2.41	0.300
PC-PTSD-5	31.4	41.9	331.50	0.007	103.7	97.6	112.7	3.61	0.165
SASRQ	31.8	41.1	349.00	0.077	111.0	80.0	114.2	8.99	0.011
Re-experience	32.1	40.2	366.00	0.123	110.1	83.7	113.4	6.77	0.034
Avoidance	32.3	39.7	376.00	0.161	111.6	82.4	112.8	7.43	0.024
Arousal	31.6	41.4	342.00	0.062	108.9	83.1	114.5	7.30	0.026
Deterioration of function	31.9	40.8	353.50	0.080	116.0	77.3	111.7	11.26	0.004
Dissociation	32.6	39.0	390.50	0.223	110.8	81.0	113.9	8.41	0.015
SF-36									
Physical functioning	36.5	29.7	384.00	0.172	102.2	125.8	103.4	4.85	0.089
Role limitations due to physical problems	37.5	27.2	334.00	0.036	97.9	122.8	107.5	4.48	0.106
General health perceptions	36.8	27.4	337.50	0.069	99.1	116.6	107.8	2.13	0.345
Vitality	36.1	30.8	405.50	0.317	90.3	135.0	108.2	13.22	0.001
Social functioning	37.7	26.8	0	0.031	99.9	125.8	105.0	4.76	0.093
Role limitations due to emotional problems	38.4	25.2	293.00	0.009	89.7	124.9	108.3	9.07	0.011
General mental health	38.6	24.6	281.50	0.007	97.2	116.8	110.1	3.03	0.220
Changes in health	34.5	34.4	478.50	0.978	112.1	95.9	107.6	1.95	0.377
Fear of the future									
Proximity of war	28.2	49.7	176.00	<0.001	95.3	108.1	114.6	5.54	0.063
Effects of war on self or family	29.4	46.8	233.50	<0.001	97.6	98.4	116.6	6.38	0.041
Hours exposed to war news per day	23.8	31.5	215.50	0.064	73.9	93.9	86.5	4.37	0.113

I: involuntary exposure; V: voluntary exposure; V&I: voluntary and involuntary exposure.

Pearson’s chi-squared was computed to test for differences between the two groups on war-news exposure. No relation of dependency was found between the voluntary, involuntary or mixed exposure, and the participants reporting the war in Ukraine or not reporting any event (χ^2^(2, *n* = 68) = 0.606, *p* = 0.822).

Hypothesis testing was conducted to explore the second objective of understanding how voluntary and involuntary exposure to news of the war influences psychological and physical well-being (Bonferroni correction applied). Differences were found for the acute stress SASRQ full-scale (*H*(2) = 8.99, *p* = 0.011), as well as for each subscale. In all SASRQ comparisons, participants in the group who had both voluntary and involuntary exposure consistently reported more acute stress symptoms than those with solely voluntary media exposure (*p* < 0.05). The involuntary group scored higher than the mixed exposure group in the SASRQ score (*p* = 0.037), the deterioration of functioning (*p* = 0.008) and the dissociative symptoms score (*p* = 0.048) (SASRQ subscales). Furthermore, differences were found in the use of denial as a coping strategy (*H*(2) = 7.82, *p* = 0.020) (BRIEF-COPE, Denial), in that those who were involuntarily exposed held higher scores of denial than those who had both types of exposure (*p* = 0.018).

Participants with voluntary exposure had better outcomes than those exposed involuntarily in terms of health outcomes, specifically in vitality (*H*(2) = 13.22, *p* = 0.001), and role limitations due to emotional outcomes (*H*(2) = 9.07, *p* = 0.011) (SF-36 subscales). For the question on the fear that the war might affect them or their family in the future (Fear of the Future), while the overall comparison was significant (*H*(2) = 6.38, *p* = 0.041), pairwise comparisons (Bonferroni correction) were not. Results for overall comparisons are displayed in Table 2.

Lastly, the duration of media coverage watched during the Russian-Ukrainian conflict was divided into three groups: (1) participants who watched less than 1 hour of media coverage of the war (*n* = 64), (2) who estimated watching 1 hour of media coverage (*n* = 64) and (3) those who watched more than 1 hour of war-media (*n* = 37). Differences were found in SF-36 Physical Functioning (*H*(2) = 6.98, *p* = 0.031) and fear that the war might affect them or their family in the future (*H*(2) = 8.48, *p* = 0.014) (Fear of the Future), in that those who watched more than 1 hour of war-media had functioned worse physically and had greater fear than those whose daily exposure was less than 1 hour (Bonferroni correction: *p* = 0.030 and *p* = 0.028, respectively). Summary of findings are displayed in Table 3.

**Table 3.** Summary of findings.

Variables of interest	Description of finding
Exposure to RUW (voluntary vs involuntary)	Involuntary exposure is linked to more acute stress symptoms; voluntary exposure is linked to higher vitality scores, and fewer role limitations, likely due to better emotional outcomes.
Media use as coping mechanism	Identified as a method to reduce stress and anxiety during crisis times but may lead to depression and anxiety in those voluntarily seeking information.
Effect of media-based trauma	Leads to stress, anxiety, fear, depression and negative physical health outcomes and scored lower in social functioning and mental health, higher risk of PTSD.
Fear of future war	Greater fear in those for whom the war media-based exposure was most stressful, especially in women. No significant difference between older and younger participants.
Effect on mental and physical health	Participants without stressful events scored higher in mental health and lower in physical/emotional role limitations. Media-based trauma affects mental/physical health, can increase heart problems, hypertension and make individuals more vulnerable to cardiovascular ailments.
Exposure duration to RUW	More than 1 hour per day leads to worse physical functioning and greater fear of the future. Correlated with stress levels.
Gender differences	Women reported more fear and used self-distraction as a coping strategy. Men may be less willing to admit fear.

## Discussion

In this sample, people who have involuntary access to news about the Russian-Ukrainian war seem to have more acute stress symptoms than those who voluntarily seek information about it. In this study, it was also found that this last group presented higher vitality scores and fewer role limitations, probably due to better emotional outcomes. Previous studies (Seeger et al., 2003) argue that collective trauma creates a high level of uncertainty about a severe threat. In the face of uncertainty, one’s existence is at stake and there is an innate desire to know things and to seek information so as to better understand the crisis and take appropriate actions in order to reduce the uncertainty (Lachlan et al., 2009). Additionally, a substantive body of evidence (Gans, 2004; Kubey and Peluso, 2009) has identified media use during such times as a coping mechanism, that aids in the reduction of stress and alleviates anxiety mainly through avoidant behaviours. Previous studies (Lachlan et al., 2010) also indicate that those who voluntarily sought more information reported being more depressed and anxious. Only further studies can shed light on whether (1) those who have involuntary access to war information reveal more acute stress symptoms due to the impact of that information or (2) whether those who avoid information might represent persons with avoidant behaviours due to their higher stress symptoms.

Exploring the second hypothesis, in the *SASRQ*, only 9.4% (*n* = 20) referred to the Russian-Ukrainian conflict as being the most stressful event of the past month (first group) and 22.5% (*n* = 48) said that they didn’t have any (control group). The first scored significantly lower in social functioning, and general mental health, and presented additional limitations due to physical and emotional problems. Also, these subjects used instrumental support and self-blame as preferred coping strategies. These coping strategies have been associated with a higher level of post-traumatic distress symptoms in a previous study on psychological responses to September 11 (Silver et al., 2002), which was also composed of subjects that were indirectly exposed to the 9/11 attacks and self-blame was the most significant predictor of global distress over time. In line with this data, it can be speculated in this study that the first group could be at higher risk of developing PTSD, according to the *PC-PTSD-5* measure. Even though exposure to a traumatic event through media is not sufficient to meet the DSM-V criteria for PTSD diagnosis, this type of exposure may result in post-traumatic symptoms (PTS), or reactions, as demonstrated by Houston (2009) and Pfefferbaum et al. (2019), who found a significant effect size between post-traumatic symptoms and exposure to coverage of terrorism. Entman (1989) states that mass media content affects, shapes and alters individuals’ cognitive structures. Hence, if the media captures one’s attention through the transmission of a terrorist attack or a war because this action is in direct conflict with a person’s existence and is identified as a threat by the individual, then it challenges the individual’s sense of security, and such information may generate fear and anxiety (negative symptoms). This may then lead to the formation of new trauma cognitive networks and result in PTS (Houston, 2009). More recent work (Bourne et al., 2013), demonstrates that experimental exposure to a traumatic film can activate fear circuitry in the brain and produce flashbacks – two key processes associated with the development of PTSD. Considering our findings, Media-based exposure could be researched as a candidate trigger for PTSD – while our results do not provide enough empirical evidence we believe that future studies could determine its role in contemporary understanding of trauma and stress disorders.

In what concerns fear of the future, this study’s findings support that those for whom the war media-based exposure was the most stressful event in the last month, the fear of the possibility of a war happening near them and that it might affect them or someone close to them was greater. Similarly, women revealed that they were more fearful that a war might affect them or their families and that they use self-distraction as a preferred coping strategy, which can somehow be considered a way of evading what is happening around them. Several studies (Ben-Zur et al., 2012; Nellis, 2009; Riad et al., 2022) also support the fact that female participants score higher on fear variables. It was considered the fact that whereas women could share an additional physical and social vulnerability (Warr, 1994) and consequently take more actions to protect themselves, men are socialized not to express fear and may, for this reason, be less willing to admit being fearful, as possible explanations (Sutton and Farrall, 2005). Despite these findings of a gender difference, in this sample, there wasn’t a significant difference between older and younger participants in questions regarding fear of a future war. This goes against previous evidence where younger participants are more affected (Riad et al. (2022).

Participants that didn’t report any stressful event scored higher in general mental health and lower in role limitations due to physical and emotional problems. This is in line with previous studies that showed that exposure to direct trauma (Schnurr and Green, 2004) or to media-based trauma events (Shedd et al., 2004) may affect mental and physical health. Acute stress and early psychological arousal may trigger neurobiological processes and are known as risk factors for the development of PTSD (Shalev et al., 1998). Holman et al. (2008) showed that in those exposed to television coverage of the 9/11 events, acute stress responses determined a 53% increased incidence of heart problems and physician-diagnosed hypertension 2–3 years after the event. Media appeared as a chronic reminder of the threat, prolonging the physiological arousal in some people, in this way making them more vulnerable to cardiovascular ailments (Kubzansky et al., 2007; McEwen, 2006).

In this study’s sample and in accordance with the last hypothesis, those who were exposed to the RUW for more than 1 hour per day function worse physically and have a greater fear of the future (vs those that didn’t). This is in line with previous evidence showing that stress levels are correlated with the extent of exposure where those who engage for one or more hours were nine times more likely to report high acute stress (Holman et al., 2014). Therefore, trauma seems to linger in media-based trauma-related stories, and this can prolong and encourage ruminative thinking because it constantly reminds one of human suffering (Holman et al., 2014).

## Limitations

Even though data was collected during the month of the Poland missile blast (November 2022), war was not as present in people’s daily lives as it was at the beginning of 2022, and the scores for this sample may differ to those that occurred in the first few months of the war. Indeed, studies of stress-related physiology reveal that stress-hormone responses can become less intense after repeated exposure to similar responses (Andersen et al., 2013), which may lower the scores. Also, this is a cross-sectional study and as the war develops new results could ensue – only prospective studies may determine time-framed results. Self-report psychiatric diagnoses were analysed and these would have benefited from substantiation either by adding symptom-based scales (e.g. MINI interview (REF) or evidence from clinical files). Additionally, some Brief-COPE subscales obtained low alpha values, although this might be a limitation of the scale itself rather than of the sample, as low scores have been found in other studies (e.g. Nunes et al., 2023; Pais Ribeiro and Rodrigues, 2004). Significantly, the SF-36 ‘Body-pain’ subscale was not included due to difficulties when registering the survey in Google Forms^®^. Also, without any physical pain score, pre-existing general health problems render individuals more vulnerable to acute stress symptoms could only be speculated. This study failed to explore what news format (video, images, etc.) and media type (social media networks, television, radio) were used. This information could have added meaningful density to the results. Lastly, the sample size and non-parametric testing make scores susceptible to Type II errors (Grech and Calleja, 2018).

## Conclusions

Media-based coverage of crises may entail several positive roles, yet this study shows that it actually results in several negative impacts on mental health. Subjects involuntarily exposed to news about this war had more acute stress symptoms while those with voluntary exposure were able to keep higher vitality scores and fewer limitations in their roles. Individuals more concerned about the RUW seem to be at higher risk of developing post-traumatic stress symptoms and live with a greater fear that a war might affect them or their families. Those who were more exposed to the media also evidenced a greater fear of the future, such as the female participants in this sample. These subjects tend to use a greater number of self-distraction coping strategies to deal with their worries. Finally, those subjects that reported no stress related with the RUW in the past month had better general mental health and fewer role limitations. Future large sample and prospective studies could support these exploratory results and further explore the impact of the nature of media form and coverage content. The health impact of media-based exposure to the RUW seems to be an important public health topic that could determine primary and secondary prevention policies.

## Supplemental Material

sj-docx-1-hpq-10.1177_13591053231201242 – Supplemental material for Does the media (also) keep the score? Media-based exposure to the Russian-Ukrainian war and mental health in Portugal Supplemental material, sj-docx-1-hpq-10.1177_13591053231201242 for Does the media (also) keep the score? Media-based exposure to the Russian-Ukrainian war and mental health in Portugal by Marta Castro, Joana Aires Dias and Luis Madeira in Journal of Health Psychology

sj-pdf-2-hpq-10.1177_13591053231201242 – Supplemental material for Does the media (also) keep the score? Media-based exposure to the Russian-Ukrainian war and mental health in Portugal Supplemental material, sj-pdf-2-hpq-10.1177_13591053231201242 for Does the media (also) keep the score? Media-based exposure to the Russian-Ukrainian war and mental health in Portugal by Marta Castro, Joana Aires Dias and Luis Madeira in Journal of Health Psychology

sj-pdf-3-hpq-10.1177_13591053231201242 – Supplemental material for Does the media (also) keep the score? Media-based exposure to the Russian-Ukrainian war and mental health in Portugal Supplemental material, sj-pdf-3-hpq-10.1177_13591053231201242 for Does the media (also) keep the score? Media-based exposure to the Russian-Ukrainian war and mental health in Portugal by Marta Castro, Joana Aires Dias and Luis Madeira in Journal of Health Psychology

sj-pdf-4-hpq-10.1177_13591053231201242 – Supplemental material for Does the media (also) keep the score? Media-based exposure to the Russian-Ukrainian war and mental health in Portugal Supplemental material, sj-pdf-4-hpq-10.1177_13591053231201242 for Does the media (also) keep the score? Media-based exposure to the Russian-Ukrainian war and mental health in Portugal by Marta Castro, Joana Aires Dias and Luis Madeira in Journal of Health Psychology

sj-sav-5-hpq-10.1177_13591053231201242 – Supplemental material for Does the media (also) keep the score? Media-based exposure to the Russian-Ukrainian war and mental health in Portugal Supplemental material, sj-sav-5-hpq-10.1177_13591053231201242 for Does the media (also) keep the score? Media-based exposure to the Russian-Ukrainian war and mental health in Portugal by Marta Castro, Joana Aires Dias and Luis Madeira in Journal of Health Psychology

sj-sps-6-hpq-10.1177_13591053231201242 – Supplemental material for Does the media (also) keep the score? Media-based exposure to the Russian-Ukrainian war and mental health in Portugal Supplemental material, sj-sps-6-hpq-10.1177_13591053231201242 for Does the media (also) keep the score? Media-based exposure to the Russian-Ukrainian war and mental health in Portugal by Marta Castro, Joana Aires Dias and Luis Madeira in Journal of Health Psychology

sj-spv-7-hpq-10.1177_13591053231201242 – Supplemental material for Does the media (also) keep the score? Media-based exposure to the Russian-Ukrainian war and mental health in Portugal Supplemental material, sj-spv-7-hpq-10.1177_13591053231201242 for Does the media (also) keep the score? Media-based exposure to the Russian-Ukrainian war and mental health in Portugal by Marta Castro, Joana Aires Dias and Luis Madeira in Journal of Health Psychology
