# Supplementary material for: Does the media (also) keep the score? Media-based exposure to the Russian-Ukrainian war and mental health in Portugal
Source: J Health Psychol. 2023 Oct 15;29(13):1475–88. doi: 10.1177/13591053231201242 (PMC11538770; doi:10.1177/13591053231201242)
Supplement: sj-docx-1-hpq-10.1177_13591053231201242 – Supplemental material for Does the media (also) keep the score? Media-based exposure to the Russian-Ukrainian war and mental health in Portugal [file sj-docx-1-hpq-10.1177_13591053231201242.docx]

| **Variables** | | ***N*** | **%** |
| --- | --- | --- | --- |
| Sex | |  |  |
|  | Female | 116 | 54.5 |
|  | Male | 97 | 45.5 |
| Employment status | |  |  |
|  | Student | 87 | 41 |
|  | Employed | 116 | 54.7 |
|  | Unemployed | 4 | 1.9 |
|  | Retired | 5 | 2.4 |
| Marital status | |  |  |
|  | Single | 121 | 57.1 |
|  | Married | 76 | 35.8 |
|  | Domestic partnership | 5 | 2.4 |
|  | Divorced | 10 | 4.7 |
| Area of residence | |  |  |
|  | Lisbon and Tagus Valley | 124 | 58.5 |
|  | North | 22 | 10.4 |
|  | Centre | 23 | 10.8 |
|  | South | 6 | 2.8 |
|  | Autonomous regions of Azores and Madeira | 30 | 14.2 |
|  | Other countries | 7 | 3.3 |
| Smoking habits | |  |  |
|  | Non-smoker | 112 | 52.6 |
|  | Smokes sporadically | 39 | 18.3 |
|  | Smokes daily | 25 | 11.7 |
|  | Former smoker | 37 | 17.4 |
| Drug consumption | |  |  |
|  | Non-user | 171 | 80.3 |
|  | Former user | 25 | 11.7 |
|  | Consumes sporadically | 16 | 7.5 |
|  | Consumes regularly | 1 | 0.5 |
| Alcohol consumption | |  |  |
|  | Never drank alcohol | 25 | 11.7 |
|  | Drinks sporadically | 117 | 83.1 |
|  | Drinks daily | 5 | 2.3 |
|  | Does not drink alcohol currently | 6 | 2.8 |
| Psychiatric diagnosis (present or former) | |  |  |
|  | Yes | 28 | 13.3 |
|  | No | 183 | 86.7 |
| Specific psychiatric diagnosis | |  |  |
|  | Depression | 13 | 50.0 |
|  | Anxiety | 6 | 23.1 |
|  | Burnout | 1 | 3.8 |
|  | Obsessive-compulsive disorder | 1 | 3.8 |
|  | Panic disorder | 2 | 7.7 |
|  | Anorexia | 1 | 3.8 |
|  | Bipolar disorder | 1 | 3.8 |
|  | Attention deficit hyperactivity disorder (ADHD) | 1 | 3.8 |
