# Supplementary material for: Does the media (also) keep the score? Media-based exposure to the Russian-Ukrainian war and mental health in Portugal
Source: J Health Psychol. 2023 Oct 15;29(13):1475–88. doi: 10.1177/13591053231201242 (PMC11538770; doi:10.1177/13591053231201242)
Supplement: sj-pdf-2-hpq-10.1177_13591053231201242 – Supplemental material for Does the media (also) keep the score? Media-based exposure to the Russian-Ukrainian war and mental health in Portugal [file sj-pdf-2-hpq-10.1177_13591053231201242.pdf]

Files/material

Together with this explanatory memo, the following files are attached:

- *Data set*: the data set utilized in the present study to conduct all analysis, including all variables created *a posteriori* for descriptive analysis and hypothesis testing (psychological measures and group variables). This data set is in *.sav* format.
- *Output*: the log file of the output of the results reported in this study. The output is in *.spv* format.
- *Syntax*: the log file of the syntax used to conduct the analyses necessary to obtain the results reported. Syntax is present in two formats, *.sps* and *.pdf*.

Data analysis

The following analyses were conducted in the present study:

- Kolmogorov-Smirnov normality test: to test for the approximate normality of the measures.
- Descriptive statistics: means (*M*), medians (*Mdn*), standard deviations (*Sd*), ranges, and Cronbach's alpha (α) were all performed regarding the variables of interest. When adequate, frequency analysis of sample size and proportion (%) were also conducted.
- Mann-Whitney U test: as the Kolmogorov-Smirnov normality tests resulted in the rejection of the null hypothesis (i.e., non-normal distribution of results), the Mann-Whitney U test was conducted when comparing between two groups.
- Kruskal-Wallis H test: as the Kolmogorov-Smirnov normality tests resulted in the rejection of the null hypothesis (i.e., non-normal distribution of results), the Kruskal-Wallis H test was conducted when comparing between three groups. The Bonferroni correction was applied to correct for multiple comparisons.
- Pearson's Chi-Squared test: conducted when testing for independence between two categorical variables.

Software used

The *Statistical Package for the Social Sciences* (SPSS® by IBM®) was used to conduct all statistical analysis.
