# Supplementary material for: Does the media (also) keep the score? Media-based exposure to the Russian-Ukrainian war and mental health in Portugal
Source: J Health Psychol. 2023 Oct 15;29(13):1475–88. doi: 10.1177/13591053231201242 (PMC11538770; doi:10.1177/13591053231201242)
Supplement: sj-pdf-3-hpq-10.1177_13591053231201242 – Supplemental material for Does the media (also) keep the score? Media-based exposure to the Russian-Ukrainian war and mental health in Portugal [file sj-pdf-3-hpq-10.1177_13591053231201242.pdf]

Introduction

This cross-sectional study provides insight into the psychological impact of media-based exposure to war on individuals' mental health.

File list

In the present study, the following four files were enclosed:

1. *Data set*: the data set utilized in the present study to conduct all analysis, including all variables created *a posteriori* for descriptive analysis and hypothesis testing (psychological measures and group variables). This data set is in *.sav* format. Data is provided to enable replication.
2. *Output*: the log file of the output of the results reported in this study. The output is in .spv format. All the results reported in this study can be found and consulted in this file.
3. *Syntax*: the log file of the syntax used to conduct the analyses necessary to obtain the results reported. The syntax is present in two formats, *.sps,* and *.pdf*. In this syntax file, it is possible to verify in detail all methods and statistical techniques used to obtain the results produced in the output, and which variables were used. This syntax can additionally be employed to test if the same results are obtained in a different, albeit similar, dataset.
4. *Explanatory memo*: this *.pdf* file summarily explains the different enclosed files, describes the statistical analysis performed, and provides information on the software used.
5. *Main Document*: manuscript .pdf file with no identifying author information.
6. *Title page: a* separate title page with author details in *.pdf*.

Instructions for replication

Questionnaires should be created in Google Forms and divulgated by online social networks (Twitter, Facebook, Instagram, and LinkedIn). The scales used in this study should be validated and culturally adapted. All data should be anonymized by assigning a code to each participant which will belong to a Case Report Form – no name or email will be collected. A different file with the same protection should contain the codes associated with clinical information to assure rare situations

where it might allow us to track persons (date of birth, place of residence). All data in the CRF must be extracted from and be consistent with the relevant source documents. The CRFs must be completed, dated, and signed by the investigator or designee in a timely manner. It remains the responsibility of the investigator for the timing, completeness, legibility, and accuracy of the CRF pages. The CRF should be accessible to study coordinators, data managers, investigators, Clinical Monitors, Auditors, and Inspectors as required. To enable peer review, monitoring, audit, and/or inspection the investigator must agree to keep records of all participating patients (sufficient information to link records e.g., CRFs, clinical records, and samples), all original signed informed consent forms, and copies of the CRF pages. All investigators and site staff involved in this study must comply with the requirements of the Data Protection Act 1998 and Trust Policy about the collection, storage, processing, and disclosure of personal information and will uphold the Act's core principles. The PI of the study should ensure that only anonymized data is received by the study team. Additionally, the list of enrolled patients with decoding should be physically separated from the coded patients in the study files.

When data is collected, it should be transferred to a software for data analysis. A first descriptive statistical analysis characterizing the sample on their sociodemographic and clinical characteristics, and their scale scores should be conducted (e.g., mean, standard deviation, median, range, Cronbach's alpha). Then, tests of normality (e.g., Kolmogorov-Smirnov) should be performed to ensure the appropriate inferential tests are used. If data is normally distributed, parametric testes should be used (e.g., Student's t test, one-way ANOVA); if not, the researcher should opt for adequate non-parametric alternatives (i.e., Mann-Whitney's U test, Kruskal-Wallis H test with Bonferroni correction). When necessary, other non-parametric tests can be used, such as Pearson's chi-square. When possible, exact p-values should be provided.
