# Supplementary material for: Does the media (also) keep the score? Media-based exposure to the Russian-Ukrainian war and mental health in Portugal
Source: J Health Psychol. 2023 Oct 15;29(13):1475–88. doi: 10.1177/13591053231201242 (PMC11538770; doi:10.1177/13591053231201242)
Supplement: sj-pdf-4-hpq-10.1177_13591053231201242 – Supplemental material for Does the media (also) keep the score? Media-based exposure to the Russian-Ukrainian war and mental health in Portugal [file sj-pdf-4-hpq-10.1177_13591053231201242.pdf]

```
* Encoding: UTF-8.

DATASET ACTIVATE DataSet1.
FREQUENCIES VARIABLES=Idade
  /STATISTICS=STDDEV MINIMUM MAXIMUM MEAN MEDIAN
  /ORDER=ANALYSIS.

FREQUENCIES VARIABLES=Sexo_Género
  /ORDER=ANALYSIS.

FREQUENCIES VARIABLES=Estado_Civil
  /ORDER=ANALYSIS.

FREQUENCIES VARIABLES=Situação_Laboral
  /ORDER=ANALYSIS.

FREQUENCIES VARIABLES=Local_Residência_Categórico
  /ORDER=ANALYSIS.

FREQUENCIES VARIABLES=Álcool Tabaco Drogas
  /ORDER=ANALYSIS.

FREQUENCIES VARIABLES=Presença_Distúrbio_Psicológico
  /ORDER=ANALYSIS.

FREQUENCIES VARIABLES=Diagnóstico_Distúrbio_Psicológico_1 Diagnóstico_Distúrbio_Psicológico_2
    Diagnóstico_Distúrbio_Psicológico_3
  /ORDER=ANALYSIS.

RELIABILITY
  /VARIABLES=BRIEF_COPE_2 BRIEF_COPE_7
  /SCALE('ALL VARIABLES') ALL
  /MODEL=ALPHA
  /SUMMARY=TOTAL.

RELIABILITY
  /VARIABLES=BRIEF_COPE_14 BRIEF_COPE_25
  /SCALE('ALL VARIABLES') ALL
  /MODEL=ALPHA
  /SUMMARY=TOTAL.

RELIABILITY
  /VARIABLES=BRIEF_COPE_10 BRIEF_COPE_23
  /SCALE('ALL VARIABLES') ALL
  /MODEL=ALPHA
  /SUMMARY=TOTAL.

RELIABILITY
  /VARIABLES=BRIEF_COPE_5 BRIEF_COPE_15
  /SCALE('ALL VARIABLES') ALL
  /MODEL=ALPHA
  /SUMMARY=TOTAL.

RELIABILITY
  /VARIABLES=BRIEF_COPE_22 BRIEF_COPE_27
  /SCALE('ALL VARIABLES') ALL
  /MODEL=ALPHA
  /SUMMARY=TOTAL.

RELIABILITY
```

```
  /VARIABLES=BRIEF_COPE_12 BRIEF_COPE_17
  /SCALE('ALL VARIABLES') ALL
  /MODEL=ALPHA
  /SUMMARY=TOTAL.

RELIABILITY
  /VARIABLES=BRIEF_COPE_13 BRIEF_COPE_26
  /SCALE('ALL VARIABLES') ALL
  /MODEL=ALPHA
  /SUMMARY=TOTAL.

RELIABILITY
  /VARIABLES=BRIEF_COPE_20 BRIEF_COPE_24
  /SCALE('ALL VARIABLES') ALL
  /MODEL=ALPHA
  /SUMMARY=TOTAL.

RELIABILITY
  /VARIABLES=BRIEF_COPE_9 BRIEF_COPE_21
  /SCALE('ALL VARIABLES') ALL
  /MODEL=ALPHA
  /SUMMARY=TOTAL.

RELIABILITY
  /VARIABLES=BRIEF_COPE_3 BRIEF_COPE_8
  /SCALE('ALL VARIABLES') ALL
  /MODEL=ALPHA
  /SUMMARY=TOTAL.

RELIABILITY
  /VARIABLES=BRIEF_COPE_1 BRIEF_COPE_19
  /SCALE('ALL VARIABLES') ALL
  /MODEL=ALPHA
  /SUMMARY=TOTAL.

RELIABILITY
  /VARIABLES=BRIEF_COPE_6 BRIEF_COPE_16
  /SCALE('ALL VARIABLES') ALL
  /MODEL=ALPHA
  /SUMMARY=TOTAL.

RELIABILITY
  /VARIABLES=BRIEF_COPE_4 BRIEF_COPE_11
  /SCALE('ALL VARIABLES') ALL
  /MODEL=ALPHA
  /SUMMARY=TOTAL.

RELIABILITY
  /VARIABLES=BRIEF_COPE_18 BRIEF_COPE_28
  /SCALE('ALL VARIABLES') ALL
  /MODEL=ALPHA
  /SUMMARY=TOTAL.

RELIABILITY
  /VARIABLES=PC_PTSD5_1 PC_PTSD5_2 PC_PTSD5_3 PC_PTSD5_4 PC_PTSD5_5
  /SCALE('ALL VARIABLES') ALL
  /MODEL=ALPHA
  /SUMMARY=TOTAL.

RELIABILITY
```

```
  /VARIABLES=SASRQ_1 SASRQ_2 SASRQ_3 SASRQ_4 SASRQ_5 SASRQ_6 SASRQ_7 SASRQ_8
SASRQ_9 SASRQ_10
    SASRQ_11 SASRQ_12 SASRQ_13 SASRQ_14 SASRQ_15 SASRQ_16 SASRQ_17 SASRQ_18
SASRQ_19 SASRQ_20 SASRQ_21
    SASRQ_22 SASRQ_23 SASRQ_24 SASRQ_25 SASRQ_26 SASRQ_27 SASRQ_28 SASRQ_29
SASRQ_30
  /SCALE('ALL VARIABLES') ALL
  /MODEL=ALPHA
  /SUMMARY=TOTAL.

RELIABILITY
  /VARIABLES=SASRQ_6 SASRQ_7 SASRQ_15 SASRQ_19 SASRQ_23 SASRQ_29
  /SCALE('ALL VARIABLES') ALL
  /MODEL=ALPHA
  /SUMMARY=TOTAL.

RELIABILITY
  /VARIABLES=SASRQ_5 SASRQ_11 SASRQ_14 SASRQ_17 SASRQ_22 SASRQ_30
  /SCALE('ALL VARIABLES') ALL
  /MODEL=ALPHA
  /SUMMARY=TOTAL.

RELIABILITY
  /VARIABLES=SASRQ_1 SASRQ_2 SASRQ_8 SASRQ_12 SASRQ_21 SASRQ_27
  /SCALE('ALL VARIABLES') ALL
  /MODEL=ALPHA
  /SUMMARY=TOTAL.

RELIABILITY
  /VARIABLES=SASRQ_9 SASRQ_26
  /SCALE('ALL VARIABLES') ALL
  /MODEL=ALPHA
  /SUMMARY=TOTAL.

RELIABILITY
  /VARIABLES=SASRQ_3 SASRQ_4 SASRQ_10 SASRQ_13 SASRQ_16 SASRQ_18 SASRQ_20
SASRQ_25 SASRQ_28
  /SCALE('ALL VARIABLES') ALL
  /MODEL=ALPHA
  /SUMMARY=TOTAL.

RELIABILITY
  /VARIABLES=SF36_3a SF36_3b SF36_3c SF36_3d SF36_3e SF36_3f SF36_3g SF36_3h SF36_3i SF36_3j
  /SCALE('ALL VARIABLES') ALL
  /MODEL=ALPHA
  /SUMMARY=TOTAL.

RELIABILITY
  /VARIABLES=SF36_4a SF36_4b SF36_4c SF36_4d
  /SCALE('ALL VARIABLES') ALL
  /MODEL=ALPHA
  /SUMMARY=TOTAL.

RELIABILITY
  /VARIABLES=SF36_5a SF36_5b SF36_5c SF36_5d
  /SCALE('ALL VARIABLES') ALL
  /MODEL=ALPHA
  /SUMMARY=TOTAL.

RELIABILITY
```

```
  /VARIABLES=SF36_1_R SF36_11a SF36_11b_R SF36_11c SF36_11d_R
  /SCALE('ALL VARIABLES') ALL
  /MODEL=ALPHA
  /SUMMARY=TOTAL.

RELIABILITY
  /VARIABLES=SF36_9a_R SF36_9e_R SF36_9g SF36_9i
  /SCALE('ALL VARIABLES') ALL
  /MODEL=ALPHA
  /SUMMARY=TOTAL.

RELIABILITY
  /VARIABLES=SF36_6_R SF36_10_R
  /SCALE('ALL VARIABLES') ALL
  /MODEL=ALPHA
  /SUMMARY=TOTAL.

RELIABILITY
  /VARIABLES=SF36_9b SF36_9c SF26_9d_R SF36_9f SF36_9h_R
  /SCALE('ALL VARIABLES') ALL
  /MODEL=ALPHA
  /SUMMARY=TOTAL.

FREQUENCIES VARIABLES=Diagnóstico_PTSD_Todos
  /ORDER=ANALYSIS.

FREQUENCIES VARIABLES=SASRQ_Evento_Perturbador_Codificado
  /ORDER=ANALYSIS.

USE ALL.
COMPUTE filter_$=(SASRQ_Evento_Perturbador_Codificado ~= 0).
VARIABLE LABELS filter_$ 'SASRQ_Evento_Perturbador_Codificado ~= 0 (FILTER)'.
VALUE LABELS filter_$ 0 'Not Selected' 1 'Selected'.
FORMATS filter_$ (f1.0).
FILTER BY filter_$.
EXECUTE.

FREQUENCIES VARIABLES=SASRQ_Quantidade_Perturbador
  /STATISTICS=STDDEV MINIMUM MAXIMUM MEAN MEDIAN
  /ORDER=ANALYSIS.

FREQUENCIES VARIABLES=SASRQ_Dias_Experienciados
  /STATISTICS=STDDEV MINIMUM MAXIMUM MEAN MEDIAN
  /ORDER=ANALYSIS.

USE ALL.
COMPUTE filter_$=(SASRQ_Evento_Perturbador_Codificado = 1).
VARIABLE LABELS filter_$ 'SASRQ_Evento_Perturbador_Codificado = 1 (FILTER)'.
VALUE LABELS filter_$ 0 'Not Selected' 1 'Selected'.
FORMATS filter_$ (f1.0).
FILTER BY filter_$.
EXECUTE.

FREQUENCIES VARIABLES=SASRQ_Quantidade_Perturbador SASRQ_Dias_Experienciados
  /STATISTICS=STDDEV MINIMUM MAXIMUM MEAN MEDIAN
  /ORDER=ANALYSIS.

USE ALL.
COMPUTE filter_$=(SASRQ_Evento_Perturbador_Codificado = 2).
VARIABLE LABELS filter_$ 'SASRQ_Evento_Perturbador_Codificado = 2 (FILTER)'.
```

```
VALUE LABELS filter_$ 0 'Not Selected' 1 'Selected'.
FORMATS filter_$ (f1.0).
FILTER BY filter_$.
EXECUTE.

FREQUENCIES VARIABLES=SASRQ_Quantidade_Perturbador SASRQ_Dias_Experienciados
  /STATISTICS=STDDEV MINIMUM MAXIMUM MEAN MEDIAN
  /ORDER=ANALYSIS.

USE ALL.
COMPUTE filter_$=(SASRQ_Evento_Perturbador_Codificado = 3).
VARIABLE LABELS filter_$ 'SASRQ_Evento_Perturbador_Codificado = 3 (FILTER)'.
VALUE LABELS filter_$ 0 'Not Selected' 1 'Selected'.
FORMATS filter_$ (f1.0).
FILTER BY filter_$.
EXECUTE.

FREQUENCIES VARIABLES=SASRQ_Quantidade_Perturbador SASRQ_Dias_Experienciados
  /STATISTICS=STDDEV MINIMUM MAXIMUM MEAN MEDIAN
  /ORDER=ANALYSIS.

USE ALL.
COMPUTE filter_$=(SASRQ_Evento_Perturbador_Codificado = 4).
VARIABLE LABELS filter_$ 'SASRQ_Evento_Perturbador_Codificado = 4 (FILTER)'.
VALUE LABELS filter_$ 0 'Not Selected' 1 'Selected'.
FORMATS filter_$ (f1.0).
FILTER BY filter_$.
EXECUTE.

FREQUENCIES VARIABLES=SASRQ_Quantidade_Perturbador SASRQ_Dias_Experienciados
  /STATISTICS=STDDEV MINIMUM MAXIMUM MEAN MEDIAN
  /ORDER=ANALYSIS.

USE ALL.
COMPUTE filter_$=(SASRQ_Evento_Perturbador_Codificado = 5).
VARIABLE LABELS filter_$ 'SASRQ_Evento_Perturbador_Codificado = 5 (FILTER)'.
VALUE LABELS filter_$ 0 'Not Selected' 1 'Selected'.
FORMATS filter_$ (f1.0).
FILTER BY filter_$.
EXECUTE.

FREQUENCIES VARIABLES=SASRQ_Quantidade_Perturbador SASRQ_Dias_Experienciados
  /STATISTICS=STDDEV MINIMUM MAXIMUM MEAN MEDIAN
  /ORDER=ANALYSIS.

USE ALL.
COMPUTE filter_$=(SASRQ_Evento_Perturbador_Codificado = 6).
VARIABLE LABELS filter_$ 'SASRQ_Evento_Perturbador_Codificado = 6 (FILTER)'.
VALUE LABELS filter_$ 0 'Not Selected' 1 'Selected'.
FORMATS filter_$ (f1.0).
FILTER BY filter_$.
EXECUTE.

FREQUENCIES VARIABLES=SASRQ_Quantidade_Perturbador SASRQ_Dias_Experienciados
  /STATISTICS=STDDEV MINIMUM MAXIMUM MEAN MEDIAN
  /ORDER=ANALYSIS.

USE ALL.
COMPUTE filter_$=(SASRQ_Evento_Perturbador_Codificado = 8).
VARIABLE LABELS filter_$ 'SASRQ_Evento_Perturbador_Codificado = 8 (FILTER)'.
```

```
VALUE LABELS filter_$ 0 'Not Selected' 1 'Selected'.
FORMATS filter_$ (f1.0).
FILTER BY filter_$.
EXECUTE.

FREQUENCIES VARIABLES=SASRQ_Quantidade_Perturbador SASRQ_Dias_Experienciados
  /STATISTICS=STDDEV MINIMUM MAXIMUM MEAN MEDIAN
  /ORDER=ANALYSIS.

USE ALL.
COMPUTE filter_$=(SASRQ_Evento_Perturbador_Codificado = 9).
VARIABLE LABELS filter_$ 'SASRQ_Evento_Perturbador_Codificado = 9 (FILTER)'.
VALUE LABELS filter_$ 0 'Not Selected' 1 'Selected'.
FORMATS filter_$ (f1.0).
FILTER BY filter_$.
EXECUTE.

FREQUENCIES VARIABLES=SASRQ_Quantidade_Perturbador SASRQ_Dias_Experienciados
  /STATISTICS=STDDEV MINIMUM MAXIMUM MEAN MEDIAN
  /ORDER=ANALYSIS.

USE ALL.
COMPUTE filter_$=(SASRQ_Evento_Perturbador_Codificado = 10).
VARIABLE LABELS filter_$ 'SASRQ_Evento_Perturbador_Codificado = 10 (FILTER)'.
VALUE LABELS filter_$ 0 'Not Selected' 1 'Selected'.
FORMATS filter_$ (f1.0).
FILTER BY filter_$.
EXECUTE.

FREQUENCIES VARIABLES=SASRQ_Quantidade_Perturbador SASRQ_Dias_Experienciados
  /STATISTICS=STDDEV MINIMUM MAXIMUM MEAN MEDIAN
  /ORDER=ANALYSIS.

USE ALL.
COMPUTE filter_$=(SASRQ_Evento_Perturbador_Codificado = 11).
VARIABLE LABELS filter_$ 'SASRQ_Evento_Perturbador_Codificado = 11 (FILTER)'.
VALUE LABELS filter_$ 0 'Not Selected' 1 'Selected'.
FORMATS filter_$ (f1.0).
FILTER BY filter_$.
EXECUTE.

FREQUENCIES VARIABLES=SASRQ_Quantidade_Perturbador SASRQ_Dias_Experienciados
  /STATISTICS=STDDEV MINIMUM MAXIMUM MEAN MEDIAN
  /ORDER=ANALYSIS.

USE ALL.
COMPUTE filter_$=(SASRQ_Evento_Perturbador_Codificado = 12).
VARIABLE LABELS filter_$ 'SASRQ_Evento_Perturbador_Codificado = 12 (FILTER)'.
VALUE LABELS filter_$ 0 'Not Selected' 1 'Selected'.
FORMATS filter_$ (f1.0).
FILTER BY filter_$.
EXECUTE.

FREQUENCIES VARIABLES=SASRQ_Quantidade_Perturbador SASRQ_Dias_Experienciados
  /STATISTICS=STDDEV MINIMUM MAXIMUM MEAN MEDIAN
  /ORDER=ANALYSIS.

USE ALL.
COMPUTE filter_$=(SASRQ_Evento_Perturbador_Codificado = 13).
VARIABLE LABELS filter_$ 'SASRQ_Evento_Perturbador_Codificado = 13 (FILTER)'.
```

```
VALUE LABELS filter_$ 0 'Not Selected' 1 'Selected'.
FORMATS filter_$ (f1.0).
FILTER BY filter_$.
EXECUTE.

FREQUENCIES VARIABLES=SASRQ_Quantidade_Perturbador SASRQ_Dias_Experienciados
  /STATISTICS=STDDEV MINIMUM MAXIMUM MEAN MEDIAN
  /ORDER=ANALYSIS.

USE ALL.
COMPUTE filter_$=(SASRQ_Evento_Perturbador_Codificado = 14).
VARIABLE LABELS filter_$ 'SASRQ_Evento_Perturbador_Codificado = 14 (FILTER)'.
VALUE LABELS filter_$ 0 'Not Selected' 1 'Selected'.
FORMATS filter_$ (f1.0).
FILTER BY filter_$.
EXECUTE.

FREQUENCIES VARIABLES=SASRQ_Quantidade_Perturbador SASRQ_Dias_Experienciados
  /STATISTICS=STDDEV MINIMUM MAXIMUM MEAN MEDIAN
  /ORDER=ANALYSIS.

USE ALL.
COMPUTE filter_$=(SASRQ_Evento_Perturbador_Codificado = 15).
VARIABLE LABELS filter_$ 'SASRQ_Evento_Perturbador_Codificado = 15 (FILTER)'.
VALUE LABELS filter_$ 0 'Not Selected' 1 'Selected'.
FORMATS filter_$ (f1.0).
FILTER BY filter_$.
EXECUTE.

FREQUENCIES VARIABLES=SASRQ_Quantidade_Perturbador SASRQ_Dias_Experienciados
  /STATISTICS=STDDEV MINIMUM MAXIMUM MEAN MEDIAN
  /ORDER=ANALYSIS.

USE ALL.
COMPUTE filter_$=(SASRQ_Evento_Perturbador_Codificado = 16).
VARIABLE LABELS filter_$ 'SASRQ_Evento_Perturbador_Codificado = 16 (FILTER)'.
VALUE LABELS filter_$ 0 'Not Selected' 1 'Selected'.
FORMATS filter_$ (f1.0).
FILTER BY filter_$.
EXECUTE.

FREQUENCIES VARIABLES=SASRQ_Quantidade_Perturbador SASRQ_Dias_Experienciados
  /STATISTICS=STDDEV MINIMUM MAXIMUM MEAN MEDIAN
  /ORDER=ANALYSIS.

USE ALL.
COMPUTE filter_$=((SASRQ_Evento_Perturbador_Codificado = 7) +
(SASRQ_Evento_Perturbador_Codificado
    = 17) + (SASRQ_Evento_Perturbador_Codificado = 18) + (SASRQ_Evento_Perturbador_Codificado = 19) +
    (SASRQ_Evento_Perturbador_Codificado = 20) + (SASRQ_Evento_Perturbador_Codificado = 21)).
VARIABLE LABELS filter_$ '(SASRQ_Evento_Perturbador_Codificado = 7) + '+
    '(SASRQ_Evento_Perturbador_Codificado = 17) + (SASRQ_Evento_Perturbador_Codificado = 18) + '+
    '(SASRQ_Evento_Perturbador_Codificado = 19) + (SASRQ_Evento_Perturbador_Codificado = 20) + '+
    '(SASRQ_Evento_Per... (FILTER)'.
VALUE LABELS filter_$ 0 'Not Selected' 1 'Selected'.
FORMATS filter_$ (f1.0).
FILTER BY filter_$.
EXECUTE.

FREQUENCIES VARIABLES=SASRQ_Quantidade_Perturbador SASRQ_Dias_Experienciados
```

```
  /STATISTICS=STDDEV MINIMUM MAXIMUM MEAN MEDIAN
  /ORDER=ANALYSIS.

FILTER OFF.
USE ALL.
EXECUTE.

FREQUENCIES VARIABLES=SF36_7
  /STATISTICS=STDDEV MINIMUM MAXIMUM MEAN MEDIAN
  /ORDER=ANALYSIS.

FREQUENCIES VARIABLES=Acesso_Informação
  /STATISTICS=STDDEV MINIMUM MAXIMUM MEAN MEDIAN
  /ORDER=ANALYSIS.

EXAMINE VARIABLES=Duração_Exposição_Media_Métrico
  /COMPARE VARIABLE
  /PLOT=BOXPLOT
  /STATISTICS=NONE
  /NOTOTAL
  /MISSING=LISTWISE.

EXAMINE VARIABLES=Duração_Exposição_Final
  /COMPARE VARIABLE
  /PLOT=BOXPLOT
  /STATISTICS=NONE
  /NOTOTAL
  /MISSING=LISTWISE.

FREQUENCIES VARIABLES=Duração_Exposição_Final
  /STATISTICS=STDDEV MINIMUM MAXIMUM MEAN MEDIAN
  /ORDER=ANALYSIS.

EXAMINE VARIABLES=BRIEF_Coping_Ativo BRIEF_Planear BRIEF_Suporte_Instrumental
    BRIEF_Suporte_Emocional BRIEF_Religião BRIEF_Reinterpretação_Positiva BRIEF_Auto_Culpabilização
    BRIEF_Aceitação BRIEF_Expressão_Sentimentos BRIEF_Negação BRIEF_Auto_Distração
    BRIEF_Desinvestimento_Comportamental BRIED_Uso_Substâncias BRIEF_Humor
PC_PTSD5_Escala_Todos
    SASRQ_Escala_Completa SASRQ_Revivência SASRQ_Evitamento SASRQ_Aumento_Ativação
    SASRQ_Deterioração_Funcionamento SASRQ_Dissociativo SF36_Função_Física
SF36_Desempenho_Físico
    SF36_Saúde_Geral SF36_Vitalidade SF36_Função_Social SF36_Desempenho_Emocional
SF36_Saúde_Mental
    SF36_Mudança_Saúde VPRS_1 VPRS_2 Duração_Exposição_Final SASRQ_Quantidade_Perturbador
  /PLOT BOXPLOT HISTOGRAM NPPLOT
  /COMPARE GROUPS
  /STATISTICS DESCRIPTIVES
  /CINTERVAL 95
  /MISSING LISTWISE
  /NOTOTAL.

RECODE Idade (Lowest thru 24=1) (25 thru 50=2) (51 thru Highest=3) INTO Idade_Categórica.
EXECUTE.

FREQUENCIES VARIABLES=BRIEF_Coping_Ativo BRIEF_Planear BRIEF_Suporte_Instrumental
    BRIEF_Suporte_Emocional BRIEF_Religião BRIEF_Reinterpretação_Positiva BRIEF_Auto_Culpabilização
    BRIEF_Aceitação BRIEF_Expressão_Sentimentos BRIEF_Negação BRIEF_Auto_Distração
    BRIEF_Desinvestimento_Comportamental BRIED_Uso_Substâncias BRIEF_Humor
PC_PTSD5_Escala_Todos
    SASRQ_Escala_Completa SASRQ_Revivência SASRQ_Evitamento SASRQ_Aumento_Ativação
```

```
   SASRQ_Deterioração_Funcionamento SASRQ_Dissociativo SF36_Função_Física
SF36_Desempenho_Físico
   SF36_Saúde_Geral SF36_Vitalidade SF36_Função_Social SF36_Desempenho_Emocional
SF36_Saúde_Mental
   SF36_Mudança_Saúde VPRS_1 VPRS_2
  /STATISTICS=STDDEV MINIMUM MAXIMUM MEAN MEDIAN
  /ORDER=ANALYSIS.

*Nonparametric Tests: Independent Samples.
NPTESTS
  /INDEPENDENT TEST (BRIEF_Coping_Ativo BRIEF_Planear BRIEF_Suporte_Instrumental
   BRIEF_Suporte_Emocional BRIEF_Religião BRIEF_Reinterpretação_Positiva BRIEF_Auto_Culpabilização
   BRIEF_Aceitação BRIEF_Expressão_Sentimentos BRIEF_Negação BRIEF_Auto_Distração
   BRIEF_Desinvestimento_Comportamental BRIED_Uso_Substâncias BRIEF_Humor
PC_PTSD5_Escala_Todos
   SASRQ_Escala_Completa SASRQ_Revivência SASRQ_Evitamento SASRQ_Aumento_Ativação
   SASRQ_Deterioração_Funcionamento SASRQ_Dissociativo SF36_Função_Física
SF36_Desempenho_Físico
   SF36_Saúde_Geral SF36_Vitalidade SF36_Função_Social SF36_Desempenho_Emocional
SF36_Saúde_Mental
   SF36_Mudança_Saúde VPRS_1 VPRS_2 SASRQ_Quantidade_Perturbador Duração_Exposição_Final)
GROUP
   (Idade_Categórica) KRUSKAL_WALLIS(COMPARE=PAIRWISE)
  /MISSING SCOPE=ANALYSIS USERMISSING=EXCLUDE
  /CRITERIA ALPHA=0.05  CILEVEL=95.

NPAR TESTS
  /M-W= BRIEF_Coping_Ativo BRIEF_Planear BRIEF_Suporte_Instrumental BRIEF_Suporte_Emocional
   BRIEF_Religião BRIEF_Reinterpretação_Positiva BRIEF_Auto_Culpabilização BRIEF_Aceitação
   BRIEF_Expressão_Sentimentos BRIEF_Negação BRIEF_Auto_Distração
BRIEF_Desinvestimento_Comportamental
   BRIED_Uso_Substâncias BRIEF_Humor PC_PTSD5_Escala_Todos SASRQ_Escala_Completa
SASRQ_Revivência
   SASRQ_Evitamento SASRQ_Aumento_Ativação SASRQ_Deterioração_Funcionamento
SASRQ_Dissociativo
   SF36_Função_Física SF36_Desempenho_Físico SF36_Saúde_Geral SF36_Vitalidade SF36_Função_Social
   SF36_Desempenho_Emocional SF36_Saúde_Mental SF36_Mudança_Saúde VPRS_1 VPRS_2
   SASRQ_Quantidade_Perturbador Duração_Exposição_Final BY Sexo_Género(1 2)
  /STATISTICS=DESCRIPTIVES
  /MISSING ANALYSIS
  /METHOD=EXACT TIMER(5).

NPAR TESTS
  /M-W= BRIEF_Coping_Ativo BRIEF_Planear BRIEF_Suporte_Instrumental BRIEF_Suporte_Emocional
   BRIEF_Religião BRIEF_Reinterpretação_Positiva BRIEF_Auto_Culpabilização BRIEF_Aceitação
   BRIEF_Expressão_Sentimentos BRIEF_Negação BRIEF_Auto_Distração
BRIEF_Desinvestimento_Comportamental
   BRIED_Uso_Substâncias BRIEF_Humor PC_PTSD5_Escala_Todos SASRQ_Escala_Completa
SASRQ_Revivência
   SASRQ_Evitamento SASRQ_Aumento_Ativação SASRQ_Deterioração_Funcionamento
SASRQ_Dissociativo
   SF36_Função_Física SF36_Desempenho_Físico SF36_Saúde_Geral SF36_Vitalidade SF36_Função_Social
   SF36_Desempenho_Emocional SF36_Saúde_Mental SF36_Mudança_Saúde VPRS_1 VPRS_2
   SASRQ_Quantidade_Perturbador Duração_Exposição_Final BY SASRQ_Evento_Perturbador_Dic(0 1)
  /STATISTICS=DESCRIPTIVES
  /MISSING ANALYSIS
  /METHOD=EXACT TIMER(5).

CROSSTABS
  /TABLES=Acesso_Informação BY SASRQ_Evento_Perturbador_Dic
```

```
  /FORMAT=AVALUE TABLES
  /STATISTICS=CHISQ PHI
  /CELLS=COUNT ROW COLUMN
  /COUNT ROUND CELL
  /METHOD=EXACT TIMER(5).


*Nonparametric Tests: Independent Samples.
NPTESTS
  /INDEPENDENT TEST (BRIEF_Coping_Ativo BRIEF_Planear BRIEF_Suporte_Instrumental
    BRIEF_Suporte_Emocional BRIEF_Religião BRIEF_Reinterpretação_Positiva BRIEF_Auto_Culpabilização
    BRIEF_Aceitação BRIEF_Expressão_Sentimentos BRIEF_Negação BRIEF_Auto_Distração
    BRIEF_Desinvestimento_Comportamental BRIED_Uso_Substâncias BRIEF_Humor
PC_PTSD5_Escala_Todos
    SASRQ_Escala_Completa SASRQ_Revivência SASRQ_Evitamento SASRQ_Aumento_Ativação
    SASRQ_Deterioração_Funcionamento SASRQ_Dissociativo SF36_Função_Física
SF36_Desempenho_Físico
    SF36_Saúde_Geral SF36_Vitalidade SF36_Função_Social SF36_Desempenho_Emocional
SF36_Saúde_Mental
    SF36_Mudança_Saúde VPRS_1 VPRS_2 SASRQ_Quantidade_Perturbador Duração_Exposição_Final)
GROUP
    (Acesso_Informação) KRUSKAL_WALLIS(COMPARE=PAIRWISE)
  /MISSING SCOPE=ANALYSIS USERMISSING=EXCLUDE
  /CRITERIA ALPHA=0.05  CILEVEL=95.

RECODE Duração_Exposição_Final (1=2) (Lowest thru 0.99=1) (1.1 thru Highest=3) INTO
    Duração_Exposição_Três_Grupos.
EXECUTE.


*Nonparametric Tests: Independent Samples.
NPTESTS
  /INDEPENDENT TEST (BRIEF_Coping_Ativo BRIEF_Planear BRIEF_Suporte_Instrumental
    BRIEF_Suporte_Emocional BRIEF_Religião BRIEF_Reinterpretação_Positiva BRIEF_Auto_Culpabilização
    BRIEF_Aceitação BRIEF_Expressão_Sentimentos BRIEF_Negação BRIEF_Auto_Distração
    BRIEF_Desinvestimento_Comportamental BRIED_Uso_Substâncias BRIEF_Humor
PC_PTSD5_Escala_Todos
    SASRQ_Escala_Completa SASRQ_Revivência SASRQ_Evitamento SASRQ_Aumento_Ativação
    SASRQ_Deterioração_Funcionamento SASRQ_Dissociativo SF36_Função_Física
SF36_Desempenho_Físico
    SF36_Saúde_Geral SF36_Vitalidade SF36_Função_Social SF36_Desempenho_Emocional
SF36_Saúde_Mental
    SF36_Mudança_Saúde VPRS_1 VPRS_2 SASRQ_Quantidade_Perturbador Duração_Exposição_Final)
GROUP
    (Duração_Exposição_Três_Grupos) KRUSKAL_WALLIS(COMPARE=PAIRWISE)
  /MISSING SCOPE=ANALYSIS USERMISSING=EXCLUDE
  /CRITERIA ALPHA=0.05  CILEVEL=95.
```
